# Prevention of pressure ulcers at orthopaedic patients begins on the accident and emergency department

**DOI:** 10.1186/1757-7241-21-S2-A43

**Published:** 2013-09-09

**Authors:** Mai Sommer, Gitte Boier Tygesen, Anne Jacobsen

**Affiliations:** 1 Orthopaedic ward, Hospitalsenheden Horsens, Denmark; 2 Accident and Emergency Department, Hospitalsenheden Horsens, Denmark

## Background

Hospitalization causes many pressure ulcers and has important implications for the patient and the economy. Regional hospital Horsens is one of 5 hospitals in Denmark participating in ‘The Danish Safer Hospital Programme’ where one aim is to reduce and prevent pressure ulcers. The target for the hospital is to reduce pressure ulcers to less than 5% and the aim is to, asses the risk of getting pressure ulcers (screen) to 90% of the patients.

The Accident and Emergency Department (A&E) undertake the first evaluation of patients’ risk of developing pressure ulcers and this evaluation is basis for the screenings in the forward progress of the patient.

Baseline measurements showed, 4 out of 10 patients experienced pressure ulcers. The cause was identified in lack of systematic observations, documentation and individual staff members’ competence levels. A need was identified for all staff to possess the same level of knowledge about prevention and tools to support this.

## Methods

The intervention started August-2010 in the Orthopaedic ward and in January-2012 the A&E joined and consisted:

Staff members’ competencies (training and bed-side education);

Tools for systematic observation (Braden Guidelines );

Involvement of patients by providing information;

Easy access to equipment (low air fluidized beds/pillows)

All pressure ulcers of grade 1 and above were registered by nurses and validated by journal reviews and compared to the numbers of discharged patients.

## Results

Before the intervention, the percentage of the screenings showed a 40-50% occurrence of pressure ulcers at patients admitted through A&E to the Orthopaedic Ward. The study shows a decrease in amount of pressure ulcers to less than 0.5% at the Orthopaedic Ward, in addition 90% of the patients had assessed their risk.

## Conclusion

By assessing the risk of getting pressure ulcers and continue the systematic observation, it seems like the target is reached. It’s not possible to conclude that the decrease, is due solely to the intervention, this needs statistical analysis and controlled studies.

Start of screening in A&E, targeting and follow-up actions in the Orthopaedic ward do seem to reduce pressure ulcers and the message is to think progress starting in A&E.

